# Off-label use of antimicrobials among hospitalized children: a retrospective study of 3,406 patients

**DOI:** 10.3389/fmicb.2023.1173042

**Published:** 2023-05-19

**Authors:** Lin Tang, Kai Zhao, Ning Hou

**Affiliations:** ^1^Department of Pharmacy, Shandong Provincial Hospital Affiliated to Shandong First Medical University, Jinan, China; ^2^Information Network Management Office, Shandong Provincial Hospital Affiliated to Shandong First Medical University, Jinan, China; ^3^Evidence-Based Pharmacy Specialties of Shandong Pharmaceutical Association, Jinan, China

**Keywords:** off-label, pediatric, antimicrobials, inpatient, evidence-based

## Abstract

**Introduction:**

Off-label drug use is a global problem for which many countries and regions have issued legal provisions or reached an expert consensus. Off-label use is sometimes a necessity, especially since antibacterial drugs have become one of the most widely used drugs in pediatric settings and the issue of causing antimicrobial resistance has increasingly become unavoidable. It also poses additional risks, such as adverse drug reactions.

**Methods:**

Our study analyzed the antimicrobial prescriptions of pediatric inpatients in a large Chinese hospital in the first half of 2021. This retrospective investigation included 6,829 prescriptions, including 2,294 off-label prescriptions. We performed descriptive analyses of prescription antimicrobial agents among pediatric populations and reported the percentages and frequencies.

**Results:**

It was found that off-label use of antibiotics was present in many children (*n *= 1,665, 48.9%) and was most common in newborns (*n * = 328, 82.8%). Among the commonly used antibiotics in pediatric patients, cephalosporins (*n * = 2,778, 40.7%) accounted for a relatively low proportion of offlabel use (*n * = 360, 15.7%), while macrolides (*n * = 628, 27.4%) and penicillins (*n * = 610, 26.6%) accounted for a higher proportion. The off-label type mainly referred to the appropriate population (46.5%) and dosage (dose, 10.0%; frequency of administration, 48.3%).

**Discussion:**

Off-label use was due to imperfect labels, improper medications, or medication errors. Only a few consensuses could apply to pediatric patients. More clinical trials are required to update the consensus, and drug labels must be continuously improved. The prescription behavior of doctors is also needed to be regulated. Rational use of drugs, especially antimicrobials, is the responsibility of all people, including the states, medical institutions, and individuals.

## 1. Introduction

Off-label use of drugs, especially in children, is a common practice worldwide. However, the off-label use of antimicrobials can lead to irrational use and cause many problems, such as bacterial resistance, ultimately leading to fatal consequences in children due to fewer available treatment options. Several countries have issued relevant regulations on this (Zhang et al., 2012; Xie et al., 2020). Before the publication of the new medical practitioners’ law on March 1, 2022, off-label use was controversial in China. To provide a reference for clinical applications, various health professional organizations, such as the Chinese Pharmaceutical Association, Chinese Medical Association, Guangdong Province Pharmaceutical Association, and Shandong Province Pharmaceutical Association, were involved in this endeavor and issued a series of relevant specifications or expert consensuses on the off-label use of drugs. Examples of this include the Consensus on the Use of Antibacterial Drugs (Chen et al., 2015), The Consensus on Management for Off-label Drug Use in Hospitals (2014), The Catalogue of Off-label Usage (The New Usage of 2020) (2020), and the Expert Consensus on Off-Label Drug Use of Shandong Province (2021) (Hou, 2021). Continuous monitoring and improvement are essential for off-label management. We are concerned with how to practice in the clinic and improve evidence-based off-label use.

In this study, we selected the most commonly used antibiotics in pediatric patients in China. The hospital investigated in this study is a large-scale general hospital in China with more than 3,000 beds, including 300 pediatric beds, which is almost the largest in Shandong Province. We retrospectively investigated the use of off-label antibacterial drugs in the pediatric department from January to June 2021. The Expert Consensus on Off-Label Drug Use of Shandong Province (2021) (Hou, 2021) was formed through research and evidence-based evaluation, and it was referred to as the SD Consensus for clinical work. It explored the level of evidence-based clinical off-label drug use and tried to promote rational drug use.

## 2. Materials and methods

### 2.1. Data collection and collation

We conducted a retrospective chart review at a Shandong Provincial Hospital. We used the Health Information System (HIS), a non-profit and non-public administrative database, to perform a hospitalization-level drug utilization study and capture clinical and resource utilization data from the entire hospital, especially from pediatric patients.

The demographic data collected from patients in the HIS included age, sex, weight, and race. Other patient-level data included the patient’s hospitalization ID, time of the doctor’s prescription issuance, admission dates, diagnosis, prescribed drugs, dosage form, dosage strength for each charge (single dose and frequency of administration), and administration route. The major diagnostic category for each patient was assigned using the International Classification of Diseases, 10th edition (ICD-10). The prescription of one drug to one hospitalized patient was regarded as a prescription.

This study included patients less than 18 years of age who had used at least one antimicrobial agent and who were admitted between January 1, 2021, and June 30, 2021. Pediatric patients were divided into the following six age groups: (1) neonatal period (0–28 days); (2) infant period (29 days–1 year); (3) early childhood (>1–3 years); (4) preschool period (>3–6 years); (5) school-age (>6–12 years); and (6) adolescent (>12–18 years).

Off-label use (“off-label”) was determined according to the latest version of the drug insert approved by the National Medical Products Administration (2023). Different manufacturers of the same drug were evaluated according to their product instructions. National Medical Products Administration (NMPA) labels, including archived labels for revised labels, were obtained from the publicly available databases NMPA and Yaozh Data (Yaozh Data, 2023). Each prescription was reviewed and classified based on four aspects: indications, appropriate population (no pediatric information and out of the required age range), dosage (dose and frequency of administration), and usage (the administration route). Drug use was considered off-label if the administration route was changed. The drug did not include pediatric information if the patient’s age was outside the NMPA-specified age range or if the dosage was over or under the label range. Each type was recorded for multiple off-label uses in the same prescription. If the medication was not adapted to the population or usage, the dosage could not be determined. Sixty-three unique prescription antimicrobials were analyzed, representing 52 drugs based on various formulations and active pharmaceutical ingredients. According to the SD Consensus, off-label uses were marked with the appropriate level of evidence.

### 2.2. Statistical analysis

We performed descriptive analyses of prescription antimicrobial agents among pediatric populations and reported the percentages and frequencies. Continuous data were reported as the mean ± SD. The antibacterial agents were grouped by drug type according to their common name and pharmacological classification, and the percentages of off-label use were calculated according to the patient’s characteristics. All analyses were performed using Microsoft Excel 2010 and SPSS 22.0 software.

## 3. Results

### 3.1. General situation

**Figure 1:**
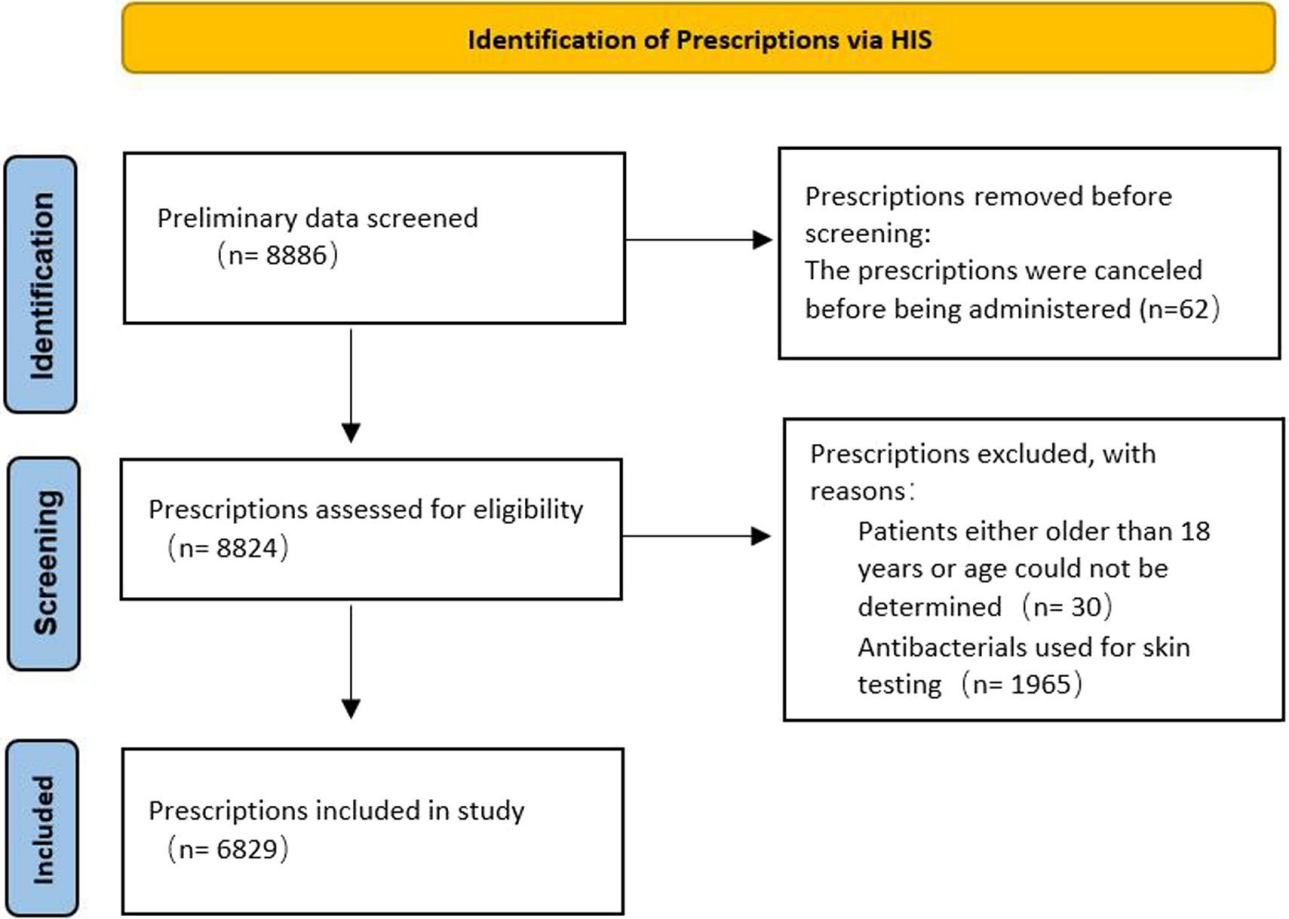
Flowchart of the included prescriptions for the study.

A total of 8,886 prescriptions were registered, but 2,057 were ineligible (Figure 1) because of age (patients either older than 18 years of age could not be determined; *n* = 30), antibacterials used for skin testing (*n* = 1,965), or the prescriptions were canceled before being administered (*n* = 62). In the first half of 2021, the total number of pediatric patients discharged from the hospital was 9,320, and 3,406 (36.5%) were treated with antibacterials. Therefore, the analysis was performed on 6,829 antibacterial prescriptions observed in 3,406 inpatients. Their demographic data and medication information are described in Supplementary Table S1. In 1,701 (49.9%) patients with a single drug prescription, 897 (26.3%) with two drugs, and 808 (23.7%) with three or more drugs, we found that the more antibacterial drugs were used, the higher the incidence of off-label use. When a patient was prescribed more than five antibacterial drugs, the incidence of off-label prescriptions exceeded 85% (Table 1). Cephalosporins were the most frequently prescribed medication, issued 2,778 times (40.7%), followed by macrolides (985 times, 14.4%), antibacterial drugs combined with enzyme inhibitors (932 times, 13.6%), carbapenems (708 times, 10.4%), and penicillins (676 times, 9.9%; Table 2). These five types of drugs were prescribed to nearly 90% (89.0%) of all pediatric inpatients treated with antimicrobials. Instead of the most frequently prescribed cephalosporins, macrolides (27.4%) and penicillins (26.6%) were mostly used off-label.

**Table 1:** Number of off-label prescription antibacterials per hospitalization case.

Number of antimicrobial prescriptions	Inpatients, *n*	Percentage of all the inpatients (*n* = 9,320)	Percentage use across the inpatients with off-label (Patients with off-label/Patients using antimicrobials, *n*)
1	1,701	18.3%	30.2% (513/1,701)
2	897	9.6%	58.3% (523/897)
3	396	4.2%	73.2% (290/396)
4	211	2.3%	79.6% (168/211)
5 or more	201	2.2%	85.1% (171/201)
Total	3,406	36.5%	48.9% (1,665/3,406)

**Table 2:** Antibacterial agents used in hospitalized children.

		Formulation	No. of off-label prescriptions/Total no. prescriptions, *n*			
		Parenteral	2,164/6,202 (34.9%)			
		Oral	130/627 (20.7%)			
Drug classification	Generic name of drug		Percentage cross all the antimicrobials prescriptions (*n* = 6,829)	No. of prescriptions according to generic name	Percentage cross all the off-label prescriptions (*n* = 2,294)	Off-label type (*n*)^#^
Cephalosporins			2,778 (40.7%)		360 (15.7%)	
	Cefuroxime	Parenteral		231	183(8.0%)	Age (19) and freq& (164)
	Cefotiam	Parenteral		50	42(1.8%)	Age (42)
	Cefaclor	Oral		208	37(1.6%)	Age (18), dose (17), and freq (2)
	Ceftizoxime	Parenteral		87	35(1.5%)	Age (33) and dose (2)
	Cefazolin	Parenteral		215	19(0.8%)	Freq (19)
	Cefaclor	Oral		16	16(0.7%)	Age (16)
	Ceftriaxone	Parenteral		1,124	12(0.5%)	Dose (7) and freq (5)
Macrolides			985 (14.4%)		628 (27.4%)	
	Azithromycin	Parenteral		568	567(24.7%)	Age (566) and dose (1)
	Azithromycin	Oral		43	15(0.7%)	Dose (13) and freq (2)
	Erythromycin	Parenteral		219	25(1.1%)	Dose (4) and freq (21)
Antibacterial drugs combined with enzyme inhibitors			932 (13.6%)		317 (13.8%)	
	Piperacillin and tazobactam	Parenteral		291	144(6.3%)	Age (140) and dose (4)
	Amoxicillin and clavulanate	Parenteral		133	123(5.4%)	Dose (4) and freq (123)
	Cefoperazone and sulbactam	Parenteral		492	45(2.0%)	Freq (45)
Carbapenems			708 (10.4%)		153 (6.7%)	
	Meropenem	Parenteral		555	148(6.5%)	Age (145), dose (2), and freq (1)
Penicillins			676 (9.9%)		610 (26.6%)	
	Flucloxacillin	Parenteral		666	608(26.5%)	Dose (113) and freq (607)
Oxazolidinones			184 (2.7%)		8 (0.3%)	
	Linezolid	Parenteral		176	6(0.3%)	Dose (3) and freq (3)
Antifungal drugs			169 (2.5%)		115 (5.0%)	
	Fluconazole	Parenteral		119	85(3.7%)	Dose (1) and freq (85)
	Micafungin	Parenteral		18	18(0.8%)	Age (18)
Glycopeptides			136 (2.0%)		5 (0.2%)	
	Vancomycin	Parenteral		115	5	Dose (5) and freq (3)
Nitroimidazoles			133 (1.9%)		16 (0.7%)	
	Ornidazole	Parenteral		75	8	Age (5), dose (2), and freq (1)
	metronidazole	Parenteral		44	6	Dose (5) and route (1)
Others			51(0.7%)	27		
	Polymyxin B Sulfate	Parenteral		12	12	Age (12)
	Gentamicin	Parenteral		10	10	Route (10)
Sulfonamides			42 (0.6%)		24 (1.0%)	
	Compound sulfamethoxazole	Oral		42	24(1.0%)	Dose (24)

^#^One prescription may have different types of off-label effects; freq&, frequency of administration.

### 3.2. Off-label use of antibacterial agents according to pediatric patient age

Off-label antimicrobials in pediatric inpatients were common in all age groups (Table 3). In this study, the off-label use of antibacterial drugs in hospitalized patients was mainly based on age, dosage, and route of administration, which was different from other studies (Carmack et al., 2020; García-López et al., 2020; Haulrig et al., 2021; San Giovanni et al., 2021), so no further analysis of the indications was performed. Among the newborns who used antimicrobials, 82.8% used them off-label. At the same time, a single patient in this age group had the most antimicrobial drug prescriptions (2.32). The frequency of off-label use of antibacterials in this age group was also the highest, at 1.99 ± 1.55. The largest number of hospitalized children were of preschool age (801), followed by 690 school-age children. The most prescribed antibacterial prescriptions were for those in preschool and early childhood (1,513 and 1,307, respectively). Age and frequency were the most frequent off-label factors, and neonatal and preschool children were the top 2. In neonates, off-label age use accounted for up to 46.8%, and off-label use of administration frequency in preschool children accounted for up to 52.7%.

**Table 3:** Off-label antimicrobial agents used according to patient age.

	Newborn	Infant	Early childhood	Preschool	School	Adolescent	Total
Total *n*. of inpatients	396	538	675	801	690	306	3,406
Total *n*. off-label inpatients	328	242	297	370	302	126	1,665
Percentage of off-label patients	82.8%	45.0%	44.0%	46.2%	43.8%	41.2%	48.9%
Total *n*. of prescriptions	920	1,249	1,307	1,513	1,282	558	6,829
Total *n*. off-label prescriptions	652	319	346	440	371	166	2,294
Percentage of off-label prescriptions	70.9%	25.5%	26.5%	29.1%	28.9%	29.7%	33.6%
Mean no. off-label prescriptions per patient ± SD	1.99 ± 1.55	1.32 ± 0.64	1.16 ± 0.43	1.19 ± 0.46	1.23 ± 0.51	1.32 ± 0.67	1.38 ± 0.89
Analysis based on the type of off-label, *n* (%)							
Age	334 (46.8%)	141 (38.8%)	162 (45.9%)	198 (44.4%)	181 (48.5%)	50 (29.9%)	1,066 (44.1%)
Dose	101 (14.2%)	36 (9.9%)	17 (4.8%)	8 (1.8%)	26 (7.0%)	41 (24.6%)	229 (9.5%)
Administration frequency	278 (39.0%)	185 (51.0%)	174 (49.3%)	235 (52.7%)	162 (43.4%)	75 (44.9%)	1,109 (45.9%)
Administration route	0 (0.0%)	1 (0.3%)	0 (0.0%)	5 (1.1%)	4 (1.1%)	1 (0.6%)	11 (0.5%)

### 3.3. Off-label use of antibacterial agents according to formulations and drugs

The vast majority of antibiotics were administered intravenously (6,202, 90.8%), and a small percentage were administered orally (627, 9.2%; Table 2). Among these, the incidence of off-label use of intravenously administered drugs was higher (34.9%). Cephalosporins were used most frequently in pediatric patients in our hospital; consistent with the results of other studies (Zhang et al., 2018), off-label use was relatively rare. Flucloxacillin, azithromycin, and antibacterial drugs combined with enzyme inhibitors are also widely used, and their off-label uses are more common.

Azithromycin had a high percentage of off-label use because its intravenous preparations were not approved for use in children under 16 years of age. The common disease for this age group was *Mycoplasma pneumonia* infection, for which the conventional treatment drug was azithromycin.

Other drugs that were used off-label due to age included meropenem (*n* = 145), piperacillin and tazobactam (*n* = 140), cefotiam (*n* = 42), and ceftizoxime (*n* = 33). Some drugs had no information on administration for children and were regarded as off-label, such as micafungin (*n* = 18), cefaclor of oral formulation (*n* = 16), and polymyxin B (*n* = 12). The main reason for off-label flucloxacillin use was the frequency (two times a day instead of four times a day), which accounted for nearly 50% of all off-label administration frequencies, followed by cefuroxime (*n* = 164), amoxicillin and clavulanate (*n* = 123), fluconazole (*n* = 85), and cefoperazone and sulbactam (*n* = 45). Penicillin and cephalosporins must be administered multiple times daily; however, this is usually not the case. Off-label fluconazole administration mainly occurred in the neonatal period, which was required to extend the interval between dosing in newborns; this is usually clinically ignored. A single dose of flucloxacillin (*n* = 113) was often not standardized; therefore, sulfamethoxazole-trimethoprim (*n* = 24) and cefaclor (*n* = 17) were used. Only two drugs were used off-label administration routes: gentamicin (10/10) and metronidazole (*n* = 1; Table 2).

Azithromycin and meropenem are recommended by the SD Consensus (Hou, 2021). Zhou et al. (2021) published recommendations on off-label use related to the intravenous administration of azithromycin in children. The off-label use of other drugs has not been recommended by expert consensus or guidelines [Chen et al., 2015; The Catalogue of Off-label Usage (The New Usage of 2020), 2020]. No severe adverse reactions were observed in this study.

## 4. Discussion

Off-label drug use is a clinically inevitable but potentially risky behavior that is more common in pediatric patients owing to the lack of clinical trials in this patient cohort (Gore et al., 2017; Shanshal and Hussain, 2021). Antibacterial agents are one of the most commonly used drugs in pediatrics, and their off-label use often causes serious social problems, such as the production of drug-resistant bacteria (European Medicines Agency, 2015; Zhang et al., 2018; Castagnola et al., 2021; Romandini et al., 2021; Yusuf and Zakir, 2021), which requires more attention. At the same time, further studies are needed on the mechanism of antimicrobial resistance (AMR), for example, “the plasmid paradox,” if it also holds true for clinically relevant AMR-harboring CPs and their bacterial hosts, which will greatly affect the measures of antibiotic resistance management (Shen et al., 2022).

This retrospective investigation included 6,829 prescriptions, including 2,294 off-label prescriptions. The main off-label factors were the appropriate population (no pediatric information and out of the required age range) and dosage (dose and frequency). We found that when more antibacterial drugs were prescribed to the same patient, the greater the possibility of off-label use; newborns had the most off-label prescriptions. Only six of the 15 most commonly used antibacterial drugs in pediatrics were evidence-based. Among them, the highest level of evidence was for meropenem, with an evidence level of one, followed by azithromycin (level 5), then other drugs (level 6). The reasons for off-label use were consistent with the results of other studies (Guo and Wang, 2014; Zhang et al., 2018; Wu and Zhou, 2020; Shanshal and Hussain, 2021). These reasons included the disease having no indications for children, the condition requiring an increase in the drug dosage or frequency of administration, and some irregular uses—insufficient single dose, less frequent administration, or changing the route of administration.

Although there are many relevant consensuses and regulations on off-label use in China, few apply to children. The SD Consensus (2022) (Tang et al., 2022) is the first to list a separate catalog for children. Although the consensus provides a vital reference basis for off-label antibacterial drugs in pediatrics, more clinical studies initiated by manufacturers or clinical investigators are also needed. Consensus and guidelines must be tracked and updated promptly.

In addition, we found many irregular uses of antibacterial drugs, which required us to strictly follow specific procedures to manage off-label use for infections at a time when the resistance rate of antibiotics is increasing significantly worldwide (Dyar et al., 2016). Most hospitals in China are restricted to off-label use when there are no reasonable alternative medicines and treatments that severely affect patients’ quality of life or cause life-threatening conditions. Adverse drug reactions, contraindications, and precautions are fully considered, and it is ensured that the usage is the best solution. Moreover, off-label use is permitted when medication is for the benefit of patients only and not for experimental research. Third, the user must be supported by advanced evidence-based medicine. Finally, the user must be reported to the Hospital Pharmaceutical Affairs Management and Pharmacotherapy Committee and the Hospital Medical Ethics Committee for approval. Informed consent should also be obtained from all the patients.

Furthermore, off-label use was not only due to imperfect labeling but also to improper medications or medication errors. The prescription behavior of doctors also needs to be regulated. Many doctors and pharmacists know about off-label drug use, but they are more concerned about the efficacy of such drugs than licensed medicines in children (Shakeel et al., 2020). Rational use of drugs, especially antimicrobials, and minimizing the risk are the responsibility of all people, including the state, medical institutions, and individuals. The state shall issue laws, norms, and management systems to regulate off-label use. The NMPA constantly urges the marketing authorization holders of these drugs to fulfill their main responsibility and requires improvements to the information presented. Associations and drug management departments should actively develop guidelines, expert consensus, and catalogs of off-label drugs. Hospitals should implement hierarchical management of off-label use, such as strict management of off-label drug use with few clinical applications, high prices, and uncertain efficacy. For a widely used off-label drug in the clinic that has a definite curative effect and a high level of evidence, NMPA should be promptly suggested to revise the label. Doctors and pharmacists should be encouraged to actively research and explore the clinical effectiveness and safety of drugs, as well as patient benefit evaluation, to provide the theoretical basis for off-label drug use.

## Data availability statement

The original contributions presented in the study are included in the article/Supplementary material, further inquiries can be directed to the corresponding author.

## Ethics statement

The studies involving human participants were reviewed and approved by Biomedical Research Ethic Committee of Shandong Provincial Hospital. Written informed consent from the participants’ legal guardian/next of kin was not required to participate in this study in accordance with the national legislation and the institutional requirements.

## Author contributions

NH, LT, and KZ conceived and designed the study. LT and KZ organized the database and performed the statistical analyses. LT wrote the first draft of the manuscript. NH wrote the sections of the manuscript. All authors contributed to the article and approved the submitted version.

## Funding

This research was supported by the Comprehensive Assessment Project of Clinical Drugs of Shandong Province No. 2021YZ022 and Bethune Medical Science Research Foundation Project No. B19258.

## Conflict of interest

The authors declare that the research was conducted without any commercial or financial relationships that could be construed as a potential conflict of interest.

## Publisher’s note

All claims expressed in this article are solely those of the authors and do not necessarily represent those of their affiliated organizations, or those of the publisher, the editors and the reviewers. Any product that may be evaluated in this article, or claim that may be made by its manufacturer, is not guaranteed or endorsed by the publisher.
